# Hemorrhoids and Their Treatment

**Published:** 1907-09

**Authors:** 


### HEMORRHOIDS AND THEIR TREATMENT.

The palliative treatment of internal hemorrhoids is often successful in the incipient stages. The exciting causes should be removed as far as possible, i. e., constipation should be relieved, any urethral, prostatic, pelvic or other diseases attended to; the diet should be restricted, and the use of condiments and stimulants should be prohibited; the external parts should be kept clean, and the patient should be kept in the recumbent position. If there is strangulation, spasm of the sphincter and pain, the application of the ice bag to the parts, application of hot water compresses or hot poultices give relief. Suppositories containing morphine, cocaine or eucaine may be used if needed. In cases where there is much hemorrhage, a thin strip of gauze moistened with weak solution of nitrate of silver (2 per cent to 4 per cent) or 10 per cent solution of ichthyol in glycerin, or 50 per cent solution of Balsan of Peru may be placed in the anal canal, where it should be allowed to remain several hours, or the bleeding points may be cauterized.

In some cases a suppository containing extract hyoscyamus gr. 2 ext. belladona gr. 1-8 to 1-4; iodoform gr. 1; oil eucalyptus gtt. 1, with or without morphine gr. 1-4 to 1-2, according to the amount of pain present, has given temporary relief, as has also an ointment containing cocaine hydrochloratis gr. iii.; sol. adrenalin chloride ʒss; bismuth sub carbonate gr. x; vaseline ℥i, applied by means of a hard rubber pile pipe morning and night and after stool.—Boston Med. Surg. Jour.

